# Phenotypic subtypes of Xia-Gibbs syndrome: a latent class analysis

**DOI:** 10.1038/s41431-024-01754-0

**Published:** 2024-12-09

**Authors:** Nan Jiang, Liyuan Zhang, Zeyan Zheng, Hanze Du, Shi Chen, Hui Pan

**Affiliations:** 1 4+4 Medical Doctor Program, Peking Union Medical College Hospital, Chinese Academy of Medical Sciences and Peking Union Medical College, 100730 Beijing, China; 2 Key Laboratory of Endocrinology of National Health Commission, Department of Endocrinology, Peking Union Medical College Hospital, Chinese Academy of Medical Sciences and Peking Union Medical College, 100730 Beijing, China

**Keywords:** Development, Growth disorders, Neurological disorders

## Abstract

Xia-Gibbs syndrome (XGS) is a rare neurodevelopmental disorder with considerable clinical heterogeneity. To further characterize the syndrome’s heterogeneity, we applied latent class analysis (LCA) on reported cases to identify phenotypic subtypes. By searching PubMed, Embase, China National Knowledge Infrastructure and Wanfang databases from inception to February 2024, we enrolled 97 cases with nonsense, frameshift or missense variants in the *AHDC1* gene. LCA was based on the following 6 phenotypes with moderate occurrence and low missingness: ataxia, seizure, autism, sleep apnea, short stature and scoliosis. After excluding cases with missing data on all LCA variables or with unmatched phenotype-genotype information, a total of 85 cases were selected for LCA. Models with 1–5 classes were compared based on Akaike Information Criterion, Bayesian Information Criterion, Sample-Size Adjusted BIC and entropy. We used multinomial logistic regression (MLR) analyses to investigate the phenotype-genotype association and potential predictors for class membership. LCA revealed 3 distinct classes labeled as Ataxia subtype (*n* = 11 [12.9%]), Sleep apnea & short stature subtype (*n* = 23 [27.1%]) and Neuropsychological subtype (*n* = 51 [60.0%]). The commonest Neuropsychological subtype was characterized by high estimated probabilities of seizure, ataxia and autism. By adjusting for sex, age and variant type, MLR showed no significant association between phenotypic subtype and variant position. Age and variant type were identified as predictors of class membership. The findings of this review offer novel insights for different presentations of XGS. It is possible to deliver targeted monitoring and treatment for each subtype in the early stage.

## Introduction

Xia-Gibbs syndrome (XGS, OMIM #615829) is a rare neurodevelopmental disorder caused by de novo autosomal dominant nonsense and frameshift variants in the AT-Hook DNA-Binding Motif-Containing 1 (*AHDC1*) gene, most of which lead to truncated protein synthesis [1, 2]. Recently, de novo missense variants in *AHDC1* have also been proposed to diagnose XGS [3, 4]. Located within the cytogenetic band 1p36.11, *AHDC1* contains one exon encoding the protein Gibbin, which plays a role in transcription, epigenetic regulation, epithelial morphogenesis and axonogenesis [5, 6]. Variable expression patterns of Gibbin have been found in the nucleoli, nucleoplasm and the whole nucleus across all tissues, but the protein is in particular highly expressed in the brain [7, 8]. The variants in *AHDC1* are postulated to alter its interaction with other proteins important for brain development, thus explaining the neurodevelopmental phenotype [9]. To date, more than 390 persons with XGS are known worldwide [10]. The disease is usually childhood-onset and has overall complex, nonspecific manifestations emerging at different ages. Core phenotypes of XGS include motor delay, speech delay, intellectual disability and hypotonia, while other features such as seizure, short stature and dysmorphisms occur less frequently [2].

The phenotypic spectrum of XGS exhibits great heterogeneity, possibly affected by a combination of demographic factors, genetic background and environmental conditions. It has been noticed that patients with XGS bearing the identical *AHDC1* variant do not necessarily have the same clinical presentation [2, 11]. Jiang et al. observed that male patients and patients with truncations near the C-terminus of Gibbin were more likely to be nonverbal [11]. The logistic regression analysis of 34 patients with XGS by Khayat et al. revealed no associations between individual phenotypes and sex, age or ethnicity; while most features could not be predicted by variant position, seizures and scoliosis were more significantly associated with truncations before the midpoint of Gibbin [2]. To better understand the disease mechanism and to promote targeted patient management, the clinical profile of XGS needs to be further characterized.

Therefore, based on reported cases with XGS in the existing literature, the leading aim of this review was to identify phenotypic subtypes of XGS by innovatively applying latent class analysis (LCA), a model-based cluster analysis approach that can be used to reveal and describe underlying patterns within a population [12]. Subsequently, we would examine the phenotype-genotype correlation and investigate predictors of class membership from candidate variables including sex, age, *AHDC1* variant type and variant position.

## Materials and methods

### Search strategy, case enrollment and data extraction

An electronic literature search was carried out in PubMed, Embase, China National Knowledge Infrastructure (CNKI) and Wanfang databases by searching for “*AHDC1*” or “Xia-Gibbs syndrome” from inception to February 2024. Search results were imported into EndNote library version X7. After removing duplicates, full-text articles were retrieved and assessed. We enrolled cases with XGS with nonsense, frameshift or missense variants in *AHDC1*; those with microdeletions or microduplications involving *AHDC1* were excluded due to the absence of substantial evidence to support their pathogenicity [13]. We also excluded cases if their specific *AHDC1* variants were not reported. Finally, a total of 97 distinct cases were enrolled from the literature.

We used a structured form in Microsoft Excel to extract data of the enrolled cases with XGS. For each individual, we recorded sex, age, *AHDC1* variant and relevant clinical features. The following phenotypes of XGS were evaluated as yes, no or not available: seizure, scoliosis, sleep apnea, ataxia, speech delay, autism, aggression, anxiety, motor delay, hypotonia, facial dysmorphism, brain dysmorphism, intellectual disability, short stature and hearing deficit. A full list of case information is outlined in Table 1.

**Table 1:** Characteristics of Xia-Gibbs syndrome cases.





1 = Yes; 0 = No.

Cases included in the cluster analysis are shadowed.

### Variable and case selection for cluster analysis

Desirable phenotypes for the cluster analysis would be those characterized by moderate occurrence and low missingness. We defined the occurrence rate as the proportion of cases exhibiting a given phenotype relative to the total number of cases in which that phenotype was reported and the missingness rate as the proportion of cases in which a given phenotype was missing relative to the total pool of 97 cases. For each phenotype under consideration, these two metrics were calculated among the 97 cases (Supplementary Table S1) and visualized using a scatter plot (Supplementary Fig. S1), which showed that ataxia, seizure, autism, sleep apnea, short stature and scoliosis were suitable variables to be included in the analysis.

We excluded cases if none of the 6 phenotypes were reported; we also removed several cases presented in conference abstracts due to the inability to accurately match genotype to phenotype data. A total of 85 cases were eventually selected for the subsequent analysis. Their missingness of the 6 phenotypes was reported as follows: ataxia(25/85, 29.4%), seizure (27/85, 31.8%), autism(18/85, 21.2%), sleep apnea(19/85, 22.4%), short stature(18/85, 21.2%) and scoliosis(25/85, 29.4%).

### Statistical analysis

LCA was applied to identify mutually exclusive XGS phenotypic subtypes based on the aforementioned 6 phenotypes, which were all coded as binary variables. Missing data were handled by Full Information Maximum Likelihood estimation, a robust method using all available data to estimate parameters without the need for imputation [14]. We performed LCA with 1–5 classes and assessed model fit using Akaike Information Criterion (AIC), Bayesian Information Criterion (BIC), Sample-Size Adjusted BIC (saBIC) and entropy. While lower information criteria (IC) values are favored, higher entropy, taking value from 0 to 1, denotes better class separation.

After determining the optimal number of classes, individuals were assigned to a class based on their highest posterior probability. Characteristics of the identified subtypes were reported as mean ± SD for normally distributed continuous variables, medians (interquartile ranges) for non-normally distributed continuous variables and numbers (percentages) for categorical variables. Analysis of variance (ANOVA), Kruskal-Wallis test, Chi-square test and Fisher’s exact test were used to compare characteristics across subtypes. For significant comparisons, post-hoc tests with Holm correction were applied to examine pair-wise differences.

Variants were classified as upstream of AT hook domain 1, between AT hook domain 1–2, between AT hook domain 2-3 and downstream of AT hook domain 3. We performed multinomial logistic regression (MLR) analysis to calculate the association between phenotypic subtype and variant position using sex, age and variant type as covariates. We then developed a series of MLR models based on candidate variables including sex, age, variant type and variant position to investigate predicting factors of class membership. AIC and BIC were used to compare models with different covariates.

LCA, statistical tests and MLR were carried out in R software version 4.2.3 and StataMP 18. A two-tailed *P*-value < 0.05 was taken as statistical significance.

## Results

### Identification of XGS phenotypic subtypes

**Fig. 1:**
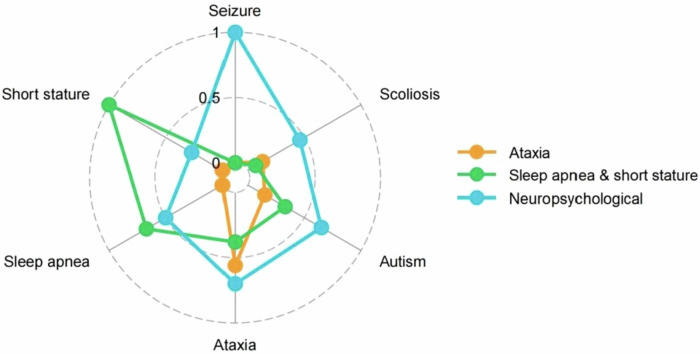
Estimated phenotype probabilities by each latent class. A radar plot visually represents class properties.

Fit indices for latent class models with 1–5 classes are shown in Supplementary Table S2. By holistically evaluating IC values and entropy, a 3-class model turned out to be the best fit. The estimated probability of the 6 phenotypes for each class is presented in Supplementary Table S3 and visualized comparatively in Fig. 1. The identified classes were labeled as Ataxia subtype, Sleep apnea & short stature subtype and Neuropsychological subtype. The Ataxia subtype was characterized by a relatively high probability of ataxia (0.56) compared to the others (0.00 ≤ probability ≤ 0.15). The Sleep apnea & short stature subtype was characterized by relatively high probabilities of sleep apnea (0.67) and short stature (1.00) compared to the others (0.00 ≤ probability ≤ 0.38). The Neuropsychological subtype was characterized by relatively high probabilities of seizure (1.00), ataxia (0.70) and autism (0.65) compared to the others (0.27 ≤ probability ≤ 0.50). Overall, 12.9% (*n* = 11) of individuals were classified into the Ataxia subtype, 27.1% (*n* = 23) into the Sleep apnea & short stature subtype and 60.0% (*n* = 51) into the Neuropsychological subtype. The missingness of the 6 phenotypes for the identified clusters is presented in Supplementary Table S4.

### Characteristics of the identified subtypes

Characteristics of the total cohort and the identified subtypes are summarized in Table 2. Observed differences across the 3 subtypes were noted for the following variables: age (*P* = 0.04), seizure (*P* < 0.001), scoliosis (*P* < 0.01), autism (*P* < 0.01), ataxia (*P* = 0.03), sleep apnea (*P* < 0.001) and short stature (*P* < 0.001). Post-hoc tests with Holm correction were applied to examine pairwise differences, which are indicated in Table 2 with different superscripts.

**Table 2:** Characteristics of the total cohort and the 3 subtypes.

Characteristic	Total	Ataxia	Sleep apnea & short stature	Neuropsychological (*n* = 51)	*P*-value
	(*n* = 85)	(*n* = 11)	(*n* = 23)		
Sex: male, n (%)	47/85 (55.3)	5/11 (45.5)	14/23 (60.9)	28/51 (54.9)	0.74
Age, months	108 (60, 156)	95.00 ± 45.62	60 (36, 138)	120 (76, 183)	0.04^b^
Variant type:					0.10
Frameshift, *n* (%)	52 (61.2)	4 (36.4)	13 (56.5)	35 (68.6)	
Nonsense, *n* (%)	25 (29.4)	6 (54.5)	9 (39.1)	10 (19.6)	
Missense, *n* (%)	8 (9.4)	1 (9.1)	1 (4.3)	6 (11.8)	
Variant position:					0.41
Upstream of AT hook domain 1, *n* (%)	19/85 (22.4)	3/11 (27.3)	2/23 (8.7)	14/51 (27.5)	
Between AT hook domain 1-2, *n* (%)	7 (8.2)	1/11 (9.1)	1/23 (4.3)	5/51 (9.8)	
Between AT hook domain 2-3, *n* (%)	15 (17.6)	1/11 (9.1)	4/23 (17.4)	10/51 (19.6)	
Downstream of AT hook domain 3, *n* (%)	44 (51.8)	6/11 (54.5)	16/23 (69.6)	22/51 (43.1)	
Seizure, *n* (%)	33/58 (56.9)	0/10 (0.0)	0/15 (0.0)	33/33 (100.0)	<0.001^b,c^
Scoliosis, *n* (%)	18/60 (30.0)	1/9 (11.1)	1/16 (6.3)	16/35 (45.7)	<0.01^b^
Autism, *n* (%)	32/67 (47.8)	1/10 (10.0)	6/18 (33.3)	25/39 (64.1)	<0.01^c^
Ataxia, *n* (%)	35/60 (58.3)	5/10 (50.0)	6/17 (35.3)	24/33 (72.7)	0.03^b^
Sleep apnea, *n* (%)	31/66 (47.0)	0/11 (0.0)	12/19 (63.2)	19/36 (52.8)	<0.001^a,c^
Short stature, *n* (%)	28/67 (41.8)	0/10 (0.0)	20/20 (100.0)	8/37 (21.6)	<0.001^a,b^
Speech delay, *n* (%)	79/83 (95.2)	10/11 (90.9)	22/22 (100.0)	47/50 (94.0)	0.32
Motor delay, *n* (%)	79/83 (95.2)	10/11 (90.9)	22/22 (100.0)	47/50 (94.0)	0.32
Intellectual disability, *n* (%)	58/61 (95.1)	5/5 (100.0)	13/14 (92.9)	40/42 (95.2)	1.00
Facial dysmorphism, *n* (%)	75/80 (93.8)	10/10 (100.0)	19/20 (95.0)	46/50 (92.0)	1.00
Brain dysmorphism, *n* (%)	61/82 (74.4)	7/10 (70.0)	14/23 (60.9)	40/49 (81.6)	0.14
Hypotonia, *n* (%)	73/85 (85.9)	8/11 (72.7)	20/23 (87.0)	45/51 (88.2)	0.40
Hearing deficit, *n* (%)	8/17 (47.1)	0/2 (0.0)	3/5 (60.0)	5/10 (50.0)	0.62

^a^Ataxia vs. Sleep apnea & short stature.

^b^Sleep apnea & short stature vs. Neuropsychological.

^c^Ataxia vs. Neuropsychological.

To investigate the relationship between age and phenotype, the occurrence of the 6 phenotypes was calculated for the following age groups: 0–6 years (preschool), 6–12 years (school-age), 12–18 years (adolescence) and above 18 years (adulthood). Post-hoc Fisher’s exact test showed that only autism had significantly different occurrence across age (*P* = 0.05) (Supplementary Table S5).

### Association between phenotype and genotype

Distribution of variants along Gibbin by phenotypic subtype and variant type is shown in Supplementary Fig. S2. In Table 3, the phenotype-genotype association was further evaluated via MLR by controlling for sex, age and variant type. The Neuropsychological subtype was treated as the reference category as most individuals belonged to this class. Neither the Ataxia subtype nor the Sleep apnea & short stature subtype exhibited significant difference in variant position compared to the Neuropsychological subtype. The subgroup analysis by variant type was not performed due to the limited number of cases.

**Table 3:** Phenotypic subtype associated with variant position.

Ataxia vs. Neuropsychological			Sleep apnea & short stature vs. Neuropsychological		
Upstream of AT hook domain 1 vs. Downstream of AT hook domain 3 RRR (95% CI)	Between AT hook domain 1-2 vs. Downstream of AT hook domain 3 RRR (95% CI)	Between AT hook domain 2-3 vs. Downstream of AT hook domain 3 RRR (95% CI)	Upstream of AT hook domain 1 vs. Downstream of AT hook domain 3 RRR (95% CI)	Between AT hook domain 1-2 vs. Downstream of AT hook domain 3 RRR (95% CI)	Between AT hook domain 2-3 vs. Downstream of AT hook domain 3 RRR (95% CI)
1.43 (0.23, 8.99)	1.16 (0.08, 15.80)	0.42 (0.04, 4.90)	0.24 (0.04, 1.40)	0.43 (0.04, 4.56)	0.65 (0.15, 2.80)

Adjusted for sex, age and variant type.

*RRR* relative risk ratio, *CI* confidence interval.

### Predictors of class membership

**Fig. 2:**
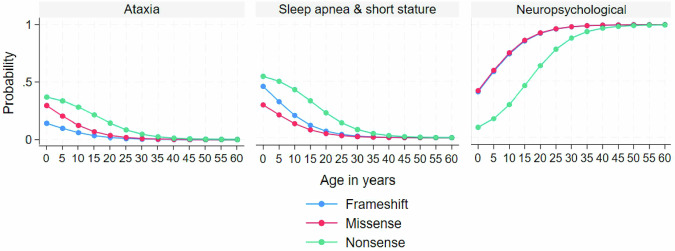
Predicted probabilities of class membership by age and variant type. The figure shows the estimated predicted probabilities of class membership from 0 to 60 years by variant type.

While the MLR model with age and variant type had the lowest AIC, the one with age alone had the lowest BIC (Supplementary Table S4). Based on theoretical considerations and expert opinions, the former model was preferred. The results are shown in Table 4. Compared to the Neuropsychological subtype, the Ataxia subtype was younger (RRR = 0.99; 95% CI [0.98, 1.00]; *P* = 0.04) and was more likely to have nonsense variants relative to frameshift variants (RRR = 12.03; 95% CI [2.22, 65.23]; *P* < 0.01). Likewise, the Sleep apnea & short stature subtype was younger (RRR = 0.99; 95% CI [0.98, 1.00]; *P* = 0.01) and was more likely to have nonsense variants relative to frameshift variants (RRR = 5.51; 95% CI [1.42, 21.47]; *P* = 0.01). Figure 2 shows the estimated predicted probability of class membership from 0 to 60 years by variant type.

**Table 4:** Multinomial logistic regression model predicting class membership.

Variable	Ataxia vs. Neuropsychological		Sleep apnea & short stature vs. Neuropsychological	
	RRR (95% CI)	*P*-value	RRR (95% CI)	*P*-value
Age, months	0.99 (0.98, 1.00)	0.04	0.99 (0.98, 1.00)	0.01
Variant type:				
Nonsense vs. frameshift	12.03 (2.22, 65.23)	<0.01	5.51 (1.42, 21.47)	0.01
Missense vs. frameshift	2.04 (0.18, 23.45)	0.57	0.62 (0.06, 6.15)	0.69

*RRR* relative risk ratio, *CI* confidence interval.

## Discussion

To our knowledge, this is the first study using LCA to identify phenotypic subtypes of XGS based on reported cases in the existing literature. Specifically, we identified 3 distinct classes: the Ataxia subtype, the Sleep apnea & short stature subtype and the Neuropsychological subtype. After adjusting for potential confounders, no significant association was observed between phenotypic subtype and *AHDC1* variant position. Age and variant type were potential predictors of class membership.

Consistent with prior research, motor delay, speech delay, intellectual disability and hypotonia are core phenotypes of XGS [2]. The LCA based on 6 phenotypes with greater variability provides novel insights into the syndrome’s heterogeneity. Patients with XGS were divided into 3 classes, each with distinct clinical features. Firstly, the Ataxia subtype was characterized by a relatively high probability of ataxia. Since ataxia is a broad term for motor incoordination, this symptom needs to be carefully followed over a long period to distinguish it from a developmental coordination disorder [15] or a cardinal feature of clumsiness among patients with Autism Spectrum Disorder [16]. An association between ataxia and posterior cranial fossa abnormalities has been observed in patients with XGS, suggesting potential brain-behavior relationships [17]. Secondly, the Sleep apnea & short stature subtype was characterized by relatively high probabilities of sleep apnea and short stature. Untreated obstructive sleep apnea can impair growth via increased energy expenditure for breathing during the night and disrupted nocturnal growth hormone (GH) secretion [18]. However, several patients with XGS presented with short stature without concurrent sleep apnea [2, 3, 11, 19–21]. This observation suggests that additional mechanisms may contribute to short stature, such as partial GH deficiency reported in 3 patients [17, 20]. Thirdly, the Neuropsychological subtype was the most prevalent class, characterized by relatively high probabilities of seizure, ataxia and autism. The frequent co-occurrence of seizure and autism is well-documented and is proposed as the result of shared divergent neurodevelopmental pathways [22]. Moreover, the mechanisms that lead to seizure may adversely affect social functioning [23].

Through cluster analysis, our review allows for a more nuanced categorization of XGS clinical presentations than previously available. Understanding the heterogeneity of this disorder is crucial for more tailored clinical management strategies. For each subtype, it is possible to implement targeted monitoring and treatment based on the estimated probability for the 6 phenotypes. Specifically, individuals in the Ataxia subtype may benefit from regular follow-ups with neurologists for assessing movement disorders [24]. Rehabilitation therapies can improve patient quality of life and safety. There are also medications that stop or slow symptom progression, but the underlying mechanisms among patients with XGS remain to be elucidated for choosing targeted therapies [25]. Individuals in the Sleep apnea & short stature subtype may benefit from regular follow-ups with otolaryngologists/sleep medicine specialists and endocrinologists for assessing sleep disturbance and retarded growth [24]. Sleep apnea can be treated with behavioral interventions, medical devices or surgical procedures [26]. For short stature, it is vital to screen for the underlying causes and initiate targeted therapies. Growth hormone deficiency, for instance, has been detected in 3 cases with XGS exhibiting good response to growth hormone replacement therapy [17, 20]. Individuals in the Neuropsychological subtype may benefit from regular follow-ups with neurologists and psychologists for assessing seizure, movement disorders and behavioral concerns so that proper.medications, rehabilitation therapies, behavioral therapies and psychiatric consultations can be delivered in time. It is also recommended to educate parents/caregivers about common seizure presentations [24].

The phenotype-genotype association has been elusive in XGS. Jiang et al. observed that patients with truncating variants closer to the C-terminal were more likely to be nonverbal and autistic [11]. The logistic regression analysis by Khayat et al. revealed no associations for most XGS features except seizure and scoliosis, which were associated with truncating variants mapping to the N-terminal to mid-protein positions [2]. These results do not align with our review, and such discrepancies may arise from different analytical perspectives. Unlike previous studies, our review does not focus on individual XGS phenotypes. Nonetheless, it has been noted that *AHDC1* variant position may not critically determine clinical presentation as patients carrying the same variant can display distinct phenotypes [11, 19]. To further establish the correlation between phenotype and variant site, a larger cohort of patients is necessary.

The 3 phenotypic subtypes of XGS differed by age and variant type. Younger age and nonsense variant were predictive of the Ataxia subtype and the Sleep apnea & short stature subtype. Our results showed that the association between older age and the Neuropsychological subtype could be explained by the late onset of autism. This pattern may reflect increased social expectations that individuals struggle to meet [17]. The association between phenotype and variant type, on the other hand, has never been reported. Khayat et al. found no associations between individual XGS features and variant type in their study of 34 patients with either frameshift or nonsense variants [2]. Although both frameshift and nonsense variants are expected to result in protein truncation, the difference in class probabilities may stem from other uncontrolled factors such as variant position, sex and ethnicity in the “optimal fit” model. Identifying predictors of XGS subtypes will empower clinicians to anticipate disease progression even before symptoms manifest, allowing for the implementation of targeted interventions in advance.

The limited number of cases has been the major obstacle for research on rare diseases. Thus, a big strength of our review is the systematic search of documented cases with XGS. While this approach alleviates the problem of sample size, inevitable limitations such as data incompleteness have to be acknowledged. To validate the present results, we recommend future research recruiting larger cohorts of patients with XGS through international collaboration and performing standardized, comprehensive data collection (including demographic information, genetic testing and phenotypic assessment). It will be possible to incorporate even more phenotypes into LCA. Prospective studies may also be considered to assess clinical outcomes among different subtypes.

In conclusion, this review validated core phenotypes of XGS based on previously reported cases. The syndrome’s heterogeneity was further elucidated through a novel application of LCA on 6 less frequently encountered phenotypes. The cluster analysis uncovered 3 phenotypic subtypes: the Ataxia subtype, the Sleep apnea & short stature subtype and the Neuropsychological subtype. The commonest class was the Neuropsychological subtype, characterized by high estimated probabilities of seizure, ataxia and autism. While no association between phenotypic subtype and *AHDC1* variant position was detected, age and variant type were potential predictors of class membership. These findings are significant, since they not only depict clinical features of XGS but also promote personalized patient care in the early stage.

## Supplementary information

Supplementary Material

## Data Availability

The data underlying this article are available in the article and its online supplementary material.
