# Correction: Phenotypic subtypes of Xia-Gibbs syndrome: a latent class analysis

**DOI:** 10.1038/s41431-025-01825-w

**Published:** 2025-04-03

**Authors:** Nan Jiang, Liyuan Zhang, Zeyan Zheng, Hanze Du, Shi Chen, Hui Pan

**Affiliations:** 1 4+4 Medical Doctor Program, Peking Union Medical College Hospital, Chinese Academy of Medical Sciences and Peking Union Medical College, Beijing, China; 2 Key Laboratory of Endocrinology of National Health Commission, Department of Endocrinology, Peking Union Medical College Hospital, Chinese Academy of Medical Sciences and Peking Union Medical College, Beijing, China

**Keywords:** Development, Growth disorders, Neurological disorders

Correction to: *European Journal of Human Genetics* 10.1038/s41431-024-01754-0, published online 09 December 2024

In this article, the second affiliation, ‘Key Laboratory of Endocrinology of National Health Commission, Department of Endocrinology, Peking Union Medical College Hospital, Chinese Academy of Medical Sciences and Peking Union Medical College, 100730, Beijing, China’ for Nan Jiang was missing.

Furthermore, the layout of Table 1 was incorrect and has been corrected. The incorrect Table 1 can be seen below:

**Table 1:** Characteristics of Xia-Gibbs syndrome cases.

ID	Publication	Nucleotide change	Protein change	Age (months)	Sex	Seizure	Scoliosis	Sleep apnea	Ataxia	Speech delay	Autism	Aggression	Anxiety	Motor delay	Hypotonia	Facial dysmorphism	Brain dysmorphism	Intellectual disability	Short stature	Hearing deficit
1	Margherita et al. [27]	c.787del	p.Ala263Profs*26	186	Female	1	0	1	1	1	1	1	1	1	1	1	1	1	0	
2	Tingting et al. [28]	c.2062C>T	p.Arg688*	38	Male															
3	Shuang-Zhu et al. [29]	c.1155dup	p.Arg386Alafs*3	6	Female	0		0		0				1	1	1	1		0	
4	Ana et al. [30]	c.2260C>T c.2062C>T			4 Males					2/4					4/4	4/4	4/4	4/4		
5																				
6		c.2641_2644dup																		
7		c.2565del																		
8	Anna et al. [31]	c.917del c.976_988del	p.Pro306Leufs*146 p.Ser326Thrfs*122						3/3	3/3		3/3		3/3	3/3	3/3	2/3	3/3	1/3	
9																				
10		c.976_988del	p.Ser326Thrfs*122																	
11	Rodrigo et al. [32]	c.1617del	p.Met539Ilefs*46	35	Female			1						1	1	1	1			
12	Ferruccio et al. [33]	c.2192dup	p.Asp732Argfs*36	84	Male	0	0	1	0	1	1			1	0	1	0	1	1	1
13		c.2188G>T	p.Glu730*	135	Male	0	0	0	0	41	0			1	0	1	1	1	0	0
14		c.4289dup	p.Ala1432Glyfs*49	80	Male	1	1	0	0	1	0			1	0	1	0	1	0	0
15		c.1446del	p.Val483Tyrfs*16	96	Male	1	0	1	0	1	1			1	1	1	1	1	0	1
16		c.1102_1114del	p.Cys368Alafs*80	112	Male	0	0	0	1	1	0			1	0	1	1	1	0	0
17	Andrea et al. [34]	c.1975del	p.Leu659Cysfs*73	132	Female	1	1			1				1	1	1	1	1		1
18		c.1975del	p.Leu659Cysfs*73	132	Female	1	1			1				1	1	1	1	1		1
19	Sumita et al. [35]	c.1206del	p.Arg403Alafs*49	120	Female	1	0	0		1	1			1	1	1	1	1	0	
20		c.1758del	p.Lys586Asnfs*3	60	Female	0	0		1	1	1			1	1	1	0	1		1
21	Michael et al. [36]	c.139C>T	p.Pro47Ser	168	Male	1	1	0	0	0	1			1	1	1	0	1	0	
22		c.1610G>A	p.Gly537Asp	120	Female	1	0	1	0	1	0			0	0	1		1	0	
23		c.1646G>A	p.Arg549His	72	Female		0	0	0	1	0			0	0	1		1	0	
24		c.1819G>A	p.Asp607Asn	276	Male	1	0	0	1	1	1			1	1		1	1	0	
25		c.2374G>C	p.Gly792Arg	144	Female	1	0	0	1	1	0			1	1	1	1	1	0	
26		c.4042T>C	p.Ser1348Pro	120	Male	1	0			1	1			1	1	1	1		0	
27		c.4432C>T	p.Pro1478Ser	132	Female	0	0	0	1	1	1			1	1	1	1	1	1	
28	Soren et al. [37]	c.2849del	p.pro950Argfs*192	120	Male	0	0	0	0	1	1	1		1	1	1	1	1	1	
29		c.2849del	p.pro950Argfs*192	132	Male	1	0	0	0	1	1	0		1	1	1	1	1	0	
30		c.2424_2425dup	p.Gly809fs*124	84	Male	0	0	0	0	1	0	0		1	1	1	1	1	0	
31		c.2188dup	p.Glu730Glyfs*38	252	Female	0	0	1	0	1	0	1		1	1	1	1	1		
32		c.2468C>G	p.Ser823*	180	Male	0	1	0	0	1	0	0		1	1	1	1	1	0	
33	Stefania et al. [38]	c.514dup	p.Ser172fs	89	Female	1	0	1	1	1	1	1		1	1	1	1	0	1	1
34	Michael et al. [39]	c.643dup	p.Ser215Lysfs*16	300	Male	1	1	1	1	0	1			1	1	1	1	1	0	
35		c.979C>T	p.Gln327*	708	Male	1	1	1	1	1	0			1	1	1	1	1	1	
36		c.1122dup	p.Gly375Argfs*3	60	Female	0	0	1	1	1	0			1	1	1	1	1	1	
37		c.1446del	p.Val483Tyrfs*16	144	Male	1	0	1	1	1	1			1	1	1	0	1	1	
38		c.1759C>T	p.Arg587*	180	Male	1	0	1	1	1	1			1	1	1		1	0	
39		c.2188G>T	p.Glu730*	144	Male	1	0	0	0	1	0			1	0	0	1	1	1	
40		c.2188G>T	p.Glu730*	144	Male	1	1	0	1	0	0			0	0	0	1	0	0	
41		c.2373_2374del	p.Cys791Trpfs*57	240	Female	1	0	1	1	1	0			1	1	1	1	1	0	
42		c.2473C>T	p.Gln825*	204	Female	1	1	1	0	1	1			1	1	1	1	1	1	
43		c.2849del	p.Pro950Argfs*192	120	Male	1	0	0	1	1	1			1	1	1	0	1	0	
44		c.2932C>T	p.Gln978*	24	Female	0	0	0	1	1	0			1	1	1	0	0	1	
45		c.3204C>G	p.Tyrl068*	36	Female	0	0	1	0	1	0			1	1	1	1	1	1	
46		c.3466C>T	p.Glnll56*	36	Male	0	0	1	1	1	0			1	1	0	0	1	1	
47		c.4438del	p.Glul480Lysfs*67	396	Female	1	0	0	0	1	0			1	1	1	1	1	0	
48	Gerrye et al. [40]	c.1122dup	p.Gly375ArgfsTer3	148	Male				1	1	1	1		1	1	1	1	1	0	
49	Augusto et al. [41]	c.451C>T	p.Arg151*	204	Male	1	1	1	1	1	1	1		1	1	1	1	1	0	
50	Ping et al. [42]	c.1122dup	p.Gly375Argfs*3	48	Female												1			
51		c.1122dup	p.Gly375Argfs*3	0	Male												1			
52	Evren [43].	c.4370A>G	p.Asp1457Gly	26	Female	1				1					1	1	1		0	
53	Lorena et al. [44]	c.1529del	p.Gly510Alafs*12	156	Female					1	1		1	0	1	1	0	1	0	0
54	Xinran et al. [45]	c.2889_2892del	p.A964fs*177	98	Male			0		1				1	1	1	1		1	
55		c.2373_2374del	p.Cys791Trpfs*57	48	Male			0		1				1	1	1	1		1	
56	David et al [46],	c.979C>T	p.Gln327*	660	Male	1	1	0	1	1		1	1	1	0	1	1	1	1	0
57	Tonne et al. [47]	c.2772del	p.Arg925Glufs	60	Male										1	1	1		1	
58	Alyssa et al. [48]	c.2473C>T	p.Gln825*	168	Female		0	1		1				1	1	1	0	1	1	0
59		c.4494dup	p.Cys1499Valfs*9	348	Male		1			1			1	1	1	1	0	1		1
60		c.2885dup	p.Pro963fs	144	Male		0			1	1			1	1	1	1	1	1	0
61		c.1759C>T	p.Arg587*	288	Male		1			1	1			1	1	1	1	1		0
62	Yunyun et al. [49]	c.2644C>T	p.Gln882*	120	Male	1	1	0	0	1	1			1	1	1	1		0	
63		c.2520del	p.Arg841Alafs*91	108	Male	0	0	0	1	1	0			1	1	1	1		0	
64		c.2773C>T	p.Arg925*	156	Male	0	1	1	0	1	0			1	1	1	0		1	
65		c.2229del	p.Ser744Profs*188	36	Male	0	0	1	0	1	0			1	1	1	0		1	
66		c.2062C>T	p.Arg688*	240	Female	1	1	0	1	1	1			1	0	1	1		0	
67		c.2908C>T	p.Gln970*	72	Female	0		0	1	1	0			1	1	1	0		0	
68		c.2691del	p.Val898Trpfs*34	48	Female	0	0	0	1	1	0			1	1		1		1	
69		c.2908C>T	p.Gln970*	132	Female	0	0	0	1	1	0			1	1		0			
70		c.784C>T	p.Gln262*	72	Female	0	0	0	0	1	1			1	1	1	0		0	
71		c.3989C>A	p.Serl330*	204	Male	0	0	0	0	1	0			1	1		0		1	
72		c.2415del	p.Leu806Trpfs*126	72	Female	1	0	0	1	1	0			1	1	1	0		0	
73		c.2773C>T	p.Arg925*	72	Female	0	0	0	1	1	0			1	1	1	1		0	
74		c.2373_2374del	p.Cys791Trpfs*57	252	Male	1	1	1	1	1	0			1	1	1	1		1	
75		c.3773C>G	p.Serl258*	24	Male	0	0	1	0	1	0			1	0		1		1	
76	Mary et al. [50],	c.2030_2030del	p.Gly677Alafs*52	96	Female			1		1				1	1	1	1	1		
77	Hui et al. [51]	c.1945del	p.Ala649Profs*83	24	Male			1	0	1	0			1	1	1	0		1	
78		c.2529_2545del	p.Asp845Argfs*40	84	Female		1		1	1	0			1	1	1	1	1	0	
79		c.1881del	p.Gln627Hisfs*105	60	Male	1			1	1	1			1	1	1	0	1	0	
80		c.1122dup	p.Gly375Argfs*3	48	Female				1	1	0			1	1	0	1	1	0	
81		c.3809del	p.Gln1270Argfs*75	108	Male				1	1	0			1	1	1	1	1	0	
82		c.2373_2374del	p.Cys791Trpfs*57	60	Male	1			1	1	0			1	1	1	1	1	0	
83		c.1480A>T	p.Lys494*	192	Female				0	1	1	1		1	0	1	1	1	1	
84	Fan et al. [52]	c.2373_2374del	p.Cys791Trpfs57	18	Female			1		1				1	1	1	1			
85		c.2898del	p.Tyr967Thrfs175	48	Female			1		1				1	1	1	1	1		
86		c.2373_2374del	p.Cys791Trpfs57	96	Male			1		1				1	1	1	1	1		
87		c.2547del	p.Ser850Profs82	132	Male			0		1	1			1	1	1	1	1		
88	Mengmeng et al. [53]	c.3543dup	p.Phe1182Valfs*11	10	Male			0		1	1			1	1	1	0		0	0
89	Yanli et al. [54]	c.1122dup	p.Gly375Argfs*3	48	Female				1	1	1			1	1	1	1	1	1	
90	Shixing et al. [55]	c.1313deI	p.Pro438fs	60						1				1	1	0	1	1		
91	Lijuan et al. [56]	c.730del	p.Ile244Serfs*16	16	Female		0	1	1	1	1			1	1	1	1	1		
92	Yingying et al. [57]	c.2136del	p.Val712fs	32	Female					1				1	1	1	1	1		
93	Huihong et al. [58]	c.4321Cå T	p.Gln1441*	7	Male			1		1				1	1	1	1	1	1	1
94		c.1122dup	p.Gly375Argfs*3	95	Female	1		1		1				1	1	0	1	1		
95	Shuting et al. [59]	c.3185_c.3186del	p.Thr1062Serfs*63	67	Male		0			1	1			1	1	1	1	1		
96	Tong et al. [60]	c.750_753del	p.Thr252Alafs*7	15	Male	0		1	0	1	0			1	1	1	1	1		
97	Kaihui et al. [61]	c.1073dup	p.Leu359Thrfs*19	25	Male		0	0		1				1	1	1	1	1		

1 = Yes; 0 = No.

Cases included in the cluster analysis are shadowed.

The correct layout for Table 1 is as shown below:

**Table 1:** Characteristics of Xia-Gibbs syndrome cases.





1 = Yes; 0 = No.

Cases included in the cluster analysis are shadowed.

The original article has been corrected.
