# Supplementary material for: Phenotypic subtypes of Xia-Gibbs syndrome: a latent class analysis
Source: Eur J Hum Genet. 2024 Dec 9;33(12):1558–66. doi: 10.1038/s41431-024-01754-0 (PMC12669642; doi:10.1038/s41431-024-01754-0)
Supplement: Supplementary file 1 — Supplementary Material [file 41431_2024_1754_MOESM1_ESM.docx]

**Supplementary Material**

**Supplementary Table S1 and Figure S1: Occurrence and missingness of phenotypes among the total pool of 97 cases.**

| **Phenotype** | **Occurrence (%)** | **Missingness (%)** |
| --- | --- | --- |
| **Seizure** | 56.9 | 40.2 |
| **Scoliosis** | 30.0 | 38.1 |
| **Sleep apnea** | 47.0 | 32.0 |
| **Ataxia** | 60.3 | 35.1 |
| **Speech delay** | 93.5 | 5.2 |
| **Autism** | 47.8 | 30.9 |
| **Aggression** | 78.6 | 85.6 |
| **Anxiety** | 100.0 | 95.9 |
| **Motor delay** | 95.5 | 9.3 |
| **Hypotonia** | 84.5 | 3.1 |
| **Facial dysmorphism** | 93.3 | 8.2 |
| **Brain dysmorphism** | 76.3 | 4.1 |
| **Intellectual disability** | 95.7 | 27.8 |
| **Short stature** | 41.4 | 27.8 |
| **Hearing deficit** | 47.1 | 82.5 |

**
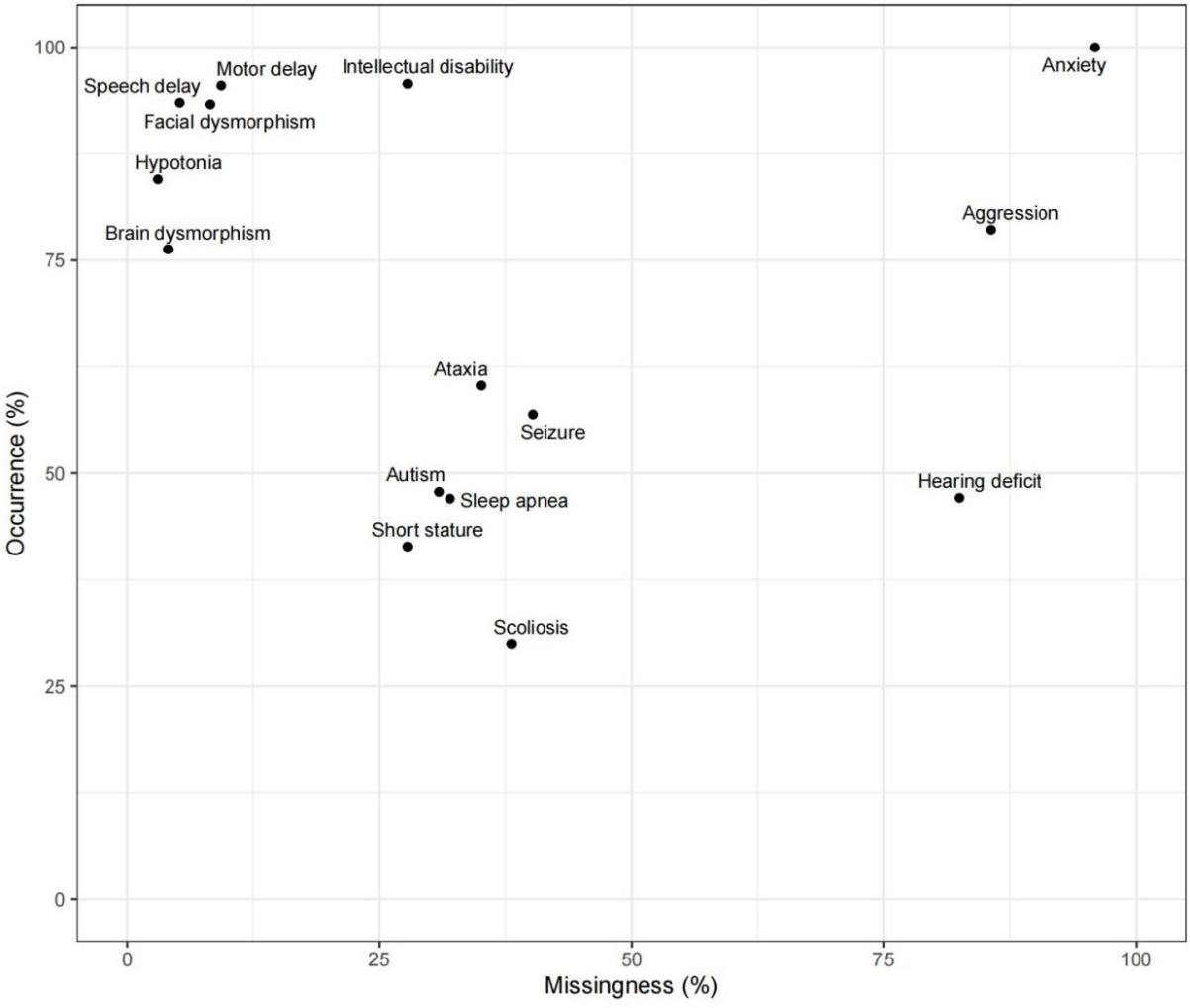
**

**Supplementary Table S2: Fit statistics for latent class models from 1 to 5 classes.**

| **Number of classes** | **Log-likelihood** | **AIC** | **BIC** | **saBIC** | **Entropy** |
| --- | --- | --- | --- | --- | --- |
| **1** | -254.59 | 521.17 | 535.83 | 516.90 | 1.00 |
| **2** | -250.03 | 526.06 | 557.81 | 516.80 | 0.80 |
| **3** | -234.20 | 508.39 | 557.24 | 494.15 | 0.82 |
| **4** | -232.14 | 518.28 | 584.23 | 499.05 | 0.78 |
| **5** | -229.16 | 526.33 | 609.38 | 502.11 | 0.69 |

AIC: Akaike Information Criterion; BIC: Bayesian Information Criterion; saBIC: Sample-Size Adjusted BIC.

**Supplementary Table S3: Estimated phenotype probabilities by each latent class.**

| **Phenotype** | **Ataxia**  **(n=11)** | **Sleep apnea**  **&**  **short stature**  **(n=23)** | **Neuropsychological**  **(n=51)** |
| --- | --- | --- | --- |
| **Seizure** | 0.00 | 0.00 | 1.00 |
| **Scoliosis** | 0.13 | 0.07 | 0.46 |
| **Autism** | 0.15 | 0.33 | 0.65 |
| **Ataxia** | 0.56 | 0.38 | 0.70 |
| **Sleep apnea** | 0.00 | 0.67 | 0.50 |
| **Short stature** | 0.00 | 1.00 | 0.27 |

**Supplementary Table S4: Missingness (%) by each latent class.**

| **Phenotype** | **Ataxia**  **(n=11)** | **Sleep apnea**  **&**  **short stature**  **(n=23)** | **Neuropsychological**  **(n=51)** |
| --- | --- | --- | --- |
| **Seizure** | 9.1 | 34.8 | 35.3 |
| **Scoliosis** | 18.2 | 30.4 | 31.4 |
| **Autism** | 9.1 | 21.7 | 23.5 |
| **Ataxia** | 9.1 | 26.1 | 35.3 |
| **Sleep apnea** | 0.0 | 17.4 | 29.4 |
| **Short stature** | 9.1 | 13.0 | 27.5 |

**Supplementary Table S5: Occurrence of the 6 phenotypes by age group.**

| **Phenotype** | **All (n=97)** | **All (n=85)** | **0-6 years (n=36)** | **6-12 years (n=31)** | **12-18 years (n=12)** | **Above 18 years (n=11)** | **P-value** |
| --- | --- | --- | --- | --- | --- | --- | --- |
| **Seizure** | 33/58 (56.9) | 33/58 (56.9) | 4/17 (23.5) | 16/24 (66.7) | 5/8 (62.5) | 8/9 (88.9) | 0.25 |
| **Scoliosis** | 18/60 (30.0) | 18/60 (30.0) | 0/15 (0.0) | 6/25 (24.0) | 5/9 (55.6) | 7/11 (63.6) | 0.08 |
| **Autism** | 32/67 (47.8) | 32/67 (47.8) | 7/22 (31.8) | 13/25 (52.0) | 8/11 (72.7) | 4/9 (44.4) | 0.05 |
| **Ataxia** | 38/63 (60.3) | 35/60 (58.3) | 13/20 (65.0) | 11/21 (52.4) | 4/10 (40.0) | 7/9 (77.8) | 0.15 |
| **Sleep apnea** | 31/66 (47.0) | 31/66 (47.0) | 12/23 (52.2) | 8/25 (32.0) | 6/9 (66.7) | 5/9 (55.6) | 0.10 |
| **Short stature** | 29/70 (41.4) | 28/67 (41.8) | 12/23 (52.2) | 8/24 (33.3) | 5/12 (41.7) | 3/8 (37.5) | 1.00 |

Number presented as n (%).

**Supplementary Figure S2: Distribution of variants along Gibbin.**

**a)**

**
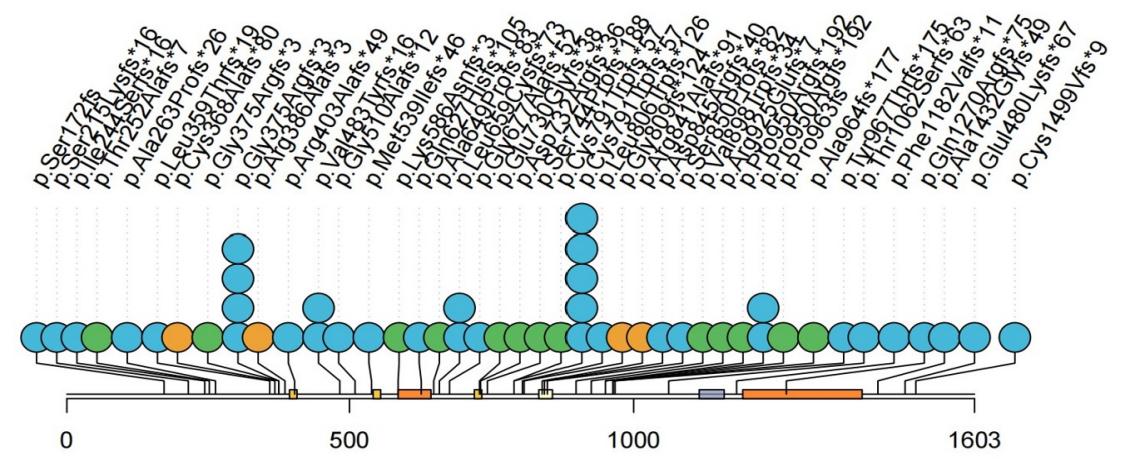
**

**b)**

**
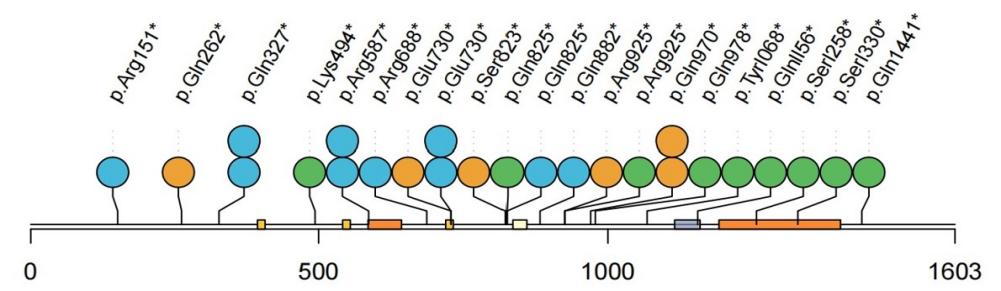
**

**c)**

**
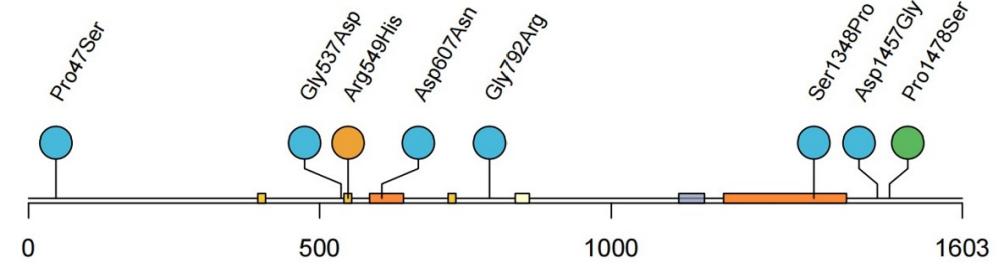
**


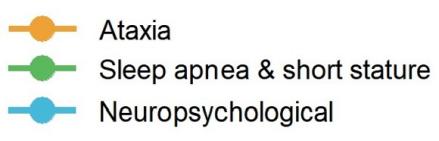

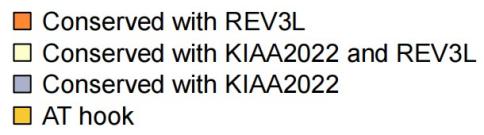


Distribution of variants by phenotypic subtype among cases with a) frameshift variants, b) nonsense variants and c) missense variants.

**Supplementary Table S6: Fit statistics for multinomial logistic regression models predicting class membership.**

| **Combination** | **AIC** | **BIC** |
| --- | --- | --- |
| **Age, sex, variant type, variant position** | 162.27 | 201.36 |
| **Age** | 156.63 | 166.40 |
| **Sex** | 164.50 | 174.27 |
| **Variant type** | 161.89 | 176.54 |
| **Variant position** | 166.66 | 186.20 |
| **Age, sex** | 159.60 | 174.26 |
| **Age, variant type** | 151.76 | 171.30 |
| **Age, variant position** | 162.73 | 187.15 |
| **Sex, variant type** | 165.15 | 184.69 |
| **Sex, variant position** | 170.21 | 194.64 |
| **Variant type, variant position** | 167.24 | 196.56 |
| **Age, sex, variant type** | 154.90 | 179.32 |
| **Age, sex, variant position** | 166.41 | 195.73 |
| **Age, variant position, variant type** | 158.40 | 192.59 |
| **Variant type, sex, variant position** | 170.77 | 204.97 |

AIC: Akaike Information Criterion; BIC: Bayesian Information Criterion.
